# Evolution of the elaborate male intromittent organ of *Xiphophorus* fishes

**DOI:** 10.1002/ece3.2396

**Published:** 2016-09-17

**Authors:** Julia C. Jones, Carmelo Fruciano, Anja Keller, Manfred Schartl, Axel Meyer

**Affiliations:** ^1^ Lehrstuhl für Zoologie und Evolutionsbiologie Department of Biology University of Konstanz Universitätstraße 10 78457 Konstanz Germany; ^2^ Zukunftskolleg University of Konstanz Konstanz Germany; ^3^ School of Earth, Environmental & Biological Sciences Queensland University of Technology Brisbane Qld 4000 Australia; ^4^ Physiological Chemistry, Biozentrum University of Würzburg Am Hubland 97074 Würzburg Germany; ^5^ Comprehensive Cancer Centre University Clinic Würzburg Josef Schneider Straße 6 97074 Würzburg Germany; ^6^ Present address: Evolution, Behaviour and Environment School of Life Sciences University of Sussex Brighton UK

**Keywords:** Male intromittent organ, reproductive character displacement, sexual selection, species diversification, *Xiphophorus* fish

## Abstract

Internally fertilizing animals show a remarkable diversity in male genital morphology that is associated with sexual selection, and these traits are thought to be evolving particularly rapidly. Male fish in some internally fertilizing species have “gonopodia,” highly modified anal fins that are putatively important for sexual selection. However, our understanding of the evolution of genital diversity remains incomplete. Contrary to the prediction that male genital traits evolve more rapidly than other traits, here we show that gonopodial traits and other nongonopodial traits exhibit similar evolutionary rates of trait change and also follow similar evolutionary models in an iconic genus of poeciliid fish (*Xiphophorus* spp.). Furthermore, we find that both mating and nonmating natural selection mechanisms are unlikely to be driving the diverse *Xiphophorus* gonopodial morphology. Putative holdfast features of the male genital organ do not appear to be influenced by water flow, a candidate selective force in aquatic habitats. Additionally, interspecific divergence in gonopodial morphology is not significantly higher between sympatric species, than between allopatric species, suggesting that male genitals have not undergone reproductive character displacement. Slower rates of evolution in gonopodial traits compared with a subset of putatively sexually selected nongenital traits suggest that different selection mechanisms may be acting on the different trait types. Further investigations of this elaborate trait are imperative to determine whether it is ultimately an important driver of speciation.

## Introduction

Genital morphology in males is generally highly variable in animals with internal fertilization, and these complex traits are thought to evolve rapidly. The variability in these traits and the potential swiftness of genital trait evolution may be explained by a number of different factors, where one of the key drivers put forward is sexual selection (Eberhard 1985, 2010a; Arnqvist 1998; Hosken and Stockley 2004; Langerhans 2011). Cryptic female choice or sexually antagonistic coevolution in particular is predicted to drive the rapid evolution of male genital morphology due to coevolution with the female (Eberhard 1996). Under cryptic female choice, females may discriminate against males (or their genitalia) before or after copulation. Sexually antagonistic selection would favor genitalia that allow males to gain control of reproduction (e.g., insemination or fertilization), and a tight coevolutionary arms race of male and female genitalia would be expected to ensue (Hosken and Stockley 2004; Klaczko et al. 2015). Natural selection mechanisms have received comparatively less attention as drivers of the evolution of diversity in male genitalia (Eberhard 1985; Arnqvist 1998; Hosken and Stockley 2004; but see Langerhans et al. 2005; Heinen Kay and Langerhans 2013; Heinen‐Kay et al. 2014). Such selective pressures can include habitat ecology, like conspicuousness or locomotor abilities in water environments, and are thought to play an important role in genital evolution in poeciliid fishes, for example (Langerhans 2011). By comparison, some species in this family of fish (genus *Xiphophorus*) with a longer sexually selected caudal fin or swordtail do not incur a cost to swimming and aerobic locomotion is not constrained (Oufiero et al. 2014a,b). One hypothesis that has been traditionally cited is that genitalia are subject to natural selection against hybridization (lock‐and‐key hypothesis), and this hypothesis is supported by the occurrence of reproductive character displacement (Langerhans 2011). There are two main mechanisms by which lock‐and‐key reproductive isolation operates (Masly 2012). The first is the classic structural lock‐and‐key mechanism where the differences in genital morphology between species directly prevent or reduce successful copulations and/or inseminations. The second is the sensory lock‐and‐key mechanism where one or both sexes perceive the differences in genital morphology and this causes behavioral or physiological responses that result in early termination of mating attempts or postcopulatory reproductive fitness problems (Masly 2012). These mechanisms are not mutually exclusive and can operate together to give rise to reproductive isolation (Masly 2012).

Although rare to date, comparative phylogenetic studies of the rates and modes of evolution of male genital versus nongenital traits are required for understanding how and why the evolution of such diversity in male genitals arises. Systems characterized by a diverse group of species that exhibit a variation in genital and also nongenital traits are key for such investigations.

**Figure 1:**
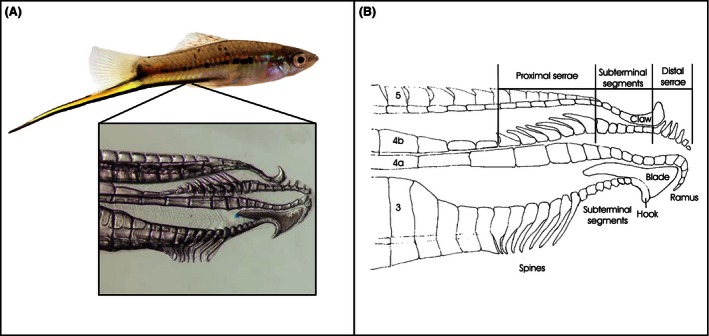
The gonopodium structure and location in an exemplar *Xiphophorus* species, *X. hellerii* (A). Schematic diagram of *X. clemenciae* gonopodial tip (B). Modified from Meyer and Schartl (2003). See Table 1 for descriptions of all gonopodial characters used in this study.

**Figure 2:**
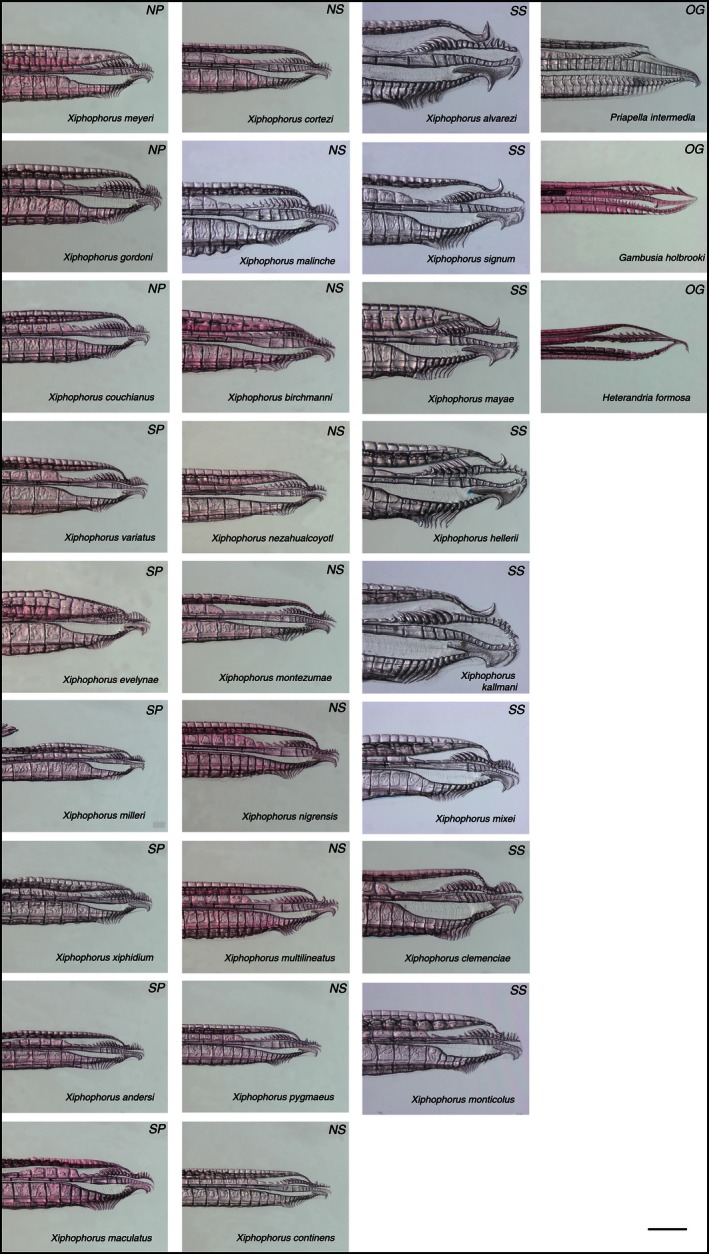
Structural diversity in gonopodial morphology of all *Xiphophorus* species. Photographs of all *Xiphophorus* species gonopodia taken after clearing and staining. Species are organized by the four main clades traditionally recognized in this genus: SS, southern swordtail; NS, northern swordtail; NP, northern platyfish; SP, southern platyfish; OG, outgroup. Scale bar represents 0.5 mm.

The genus *Xiphophorus* is comprised of 26 species of small freshwater fish called swordtails and platyfish. These fishes form a highly diverse radiation predominantly in Mexico and exhibit a large amount of variation in male genital traits (Figs. 1, 2), as well as in nongenital traits (such as the ornamental sword in males; e.g., Marcus and McCune 1999). Thus, this genus is ideal for studying the evolution of the male intromittent organ (gonopodium) as the evolutionary dynamics between diverse genital and nongenital traits can be compared. *Xiphophorus* fish are called swordtails due to the dagger‐like modified anal fins of males, some of which form the gonopodium that serves as a sperm transfer organ and is used in internal fertilization of females (Fig. 1; Heckel 1849). Females give birth to living young rather than laying eggs as in most other species of fish. Male *Xiphophorus* fish, as in other animals with internal fertilization, exhibit highly variable genital morphology (Eberhard 1985, 2010b; Edwards 1993; Hosken and Stockley 2004; Evans and Meisner 2009; Langerhans 2011). The gonopodia have been used extensively in species identification (e.g., Kallman et al. 2004). However, there is as yet no genus‐wide analysis examining the forces driving and maintaining the elaborate gonopodial morphology.

The morphology of the *Xiphophorus* male reproductive intromittent organ shows high interspecific variation through the differences in hooks, spines, claws, overall length, and other features and may be key in prezygotic isolation (Clark et al. 1954; Rosen 1979; Kallman et al. 2004; Langerhans 2011). In *Xiphophorus*, as in all poeciliids, the gonopodium develops from an undifferentiated male anal fin and is modified for transmitting spermatophores. Specifically, three elongated rays of the anal fin constitute the morphologically and functionally species‐specific distinct structure. One anal fin ray develops spines and a hook, and a second ray develops a claw‐like structure. As suggested above, different sources of both natural and, in particular, sexual selection are likely to act on genitalia (Eberhard 1985). Such sources of selection are thought to have influenced the extraordinary diversity in form seen across poeciliid fishes generally, and suggest a key role for genital diversity in speciation (Langerhans 2011). Sexual selection appears to be important in causing at least some of the observed diversity in this structure in some species of poeciliid fish (Evans et al. 2011; Kwan et al. 2013). Further, the male intromittent organ might also serve to remove previously deposited spermatophores (Eberhard 1985).

Across their distribution, from Mexico south to Honduras, *Xiphophorus* fish also show a variation in nongenital morphological traits, such as the extravagant male sword, body color, and vertical bar pigment pattern, some of which are thought to be important in mate choice (Basolo 1990; Rauchenberger et al. 1990; Morris and Casey 1998; Marcus and McCune 1999; Kingston et al. 2003). For instance, Darwin (1872) already recognized that the long colorful extensions of the ventral caudal fin, or sword, exhibited by males of some species of these fish might have arisen by sexual selection, and these longer swords have subsequently been shown to be preferred by females (Basolo 1990). Similarly, *Xiphophorus hellerii* males sporting red mid‐lateral stripes, rather than darker stripes, have been shown to be preferred by females (Franck et al. 2003), and *Xiphophorus cortezi* females have a polymorphic preference for vertical bars (Morris et al. 2003).

Here, we conducted the first study of gonopodial morphology and evolutionary dynamics that considers all 26 species of the genus *Xiphophorus*, including recently described species. We characterize and quantify gonopodial morphology and use phylogenetic comparative methods to estimate the rates of trait evolution and fit evolutionary models to determine the modes of evolution. We first examine whether the rates are faster, and whether modes differ, in gonopodial compared to nongonopodial traits. Second, we investigate whether different natural selection mechanisms, both mating (hybridization avoidance) and nonmating (habitat ecology), are playing a role in the evolution of the highly variable *Xiphophorus* gonopodial morphology.

## Methods

### Samples

The gonopodia of all 26 species of *Xiphophorus* fish were dissected from each individual, cleared with a trypsin solution, and stained using alcian blue and alizarin red (the number of individuals per species ranged from one to five, Table S1; Dingerkus and Uhler 1977). Individuals examined here are from laboratory strains bred from wild‐caught individuals. Clearing and staining was employed to ensure the clear visualization of all components of the trait. Each gonopodium was then mounted on an individual slide and photographed with a Zeiss AxioCam MRc 2 digital imaging system mounted on an M2 stereomicroscope (Zeiss, Germany) (Fig. 2).

### Trait morphology and scoring

*Xiphophorus* gonopodial morphology was characterized by scoring six different traits and using existing data for seven further traits (Fig. 1, Tables 1, S2; Marcus and McCune 1999). We also obtained data on 28 additional nongonopodial multistate characters that had been previously scored (Marcus and McCune 1999; Table 1). These include a variety of traits related to coloration, body shape, fins, and growth. We note that although such traits are known to vary extensively among *Xiphophorus* fish, very few have been identified to be under some form of selection or evolving neutrally. The gonopodial characters scored as multistate characters are known to vary between poeciliid species and especially within the genus *Xiphophorus* (Rosen 1960; Kallman et al. 2004). Here, claw presence and size were scored, and we also scored hook and ramus shape, the shape of ray 4a, and spine angle (Figs. 1, 2). Additional linear measurements were also scored to capture the fine‐scale morphology of the gonopodium when testing for ecological factors. The length of the gonopodium was measured from the anchor point of the first ray to the tip of the gonopodium.

**Table 1:** Descriptions of all gonopodial characters used in this study

Character number	Character description
Gonopodial traits	
Character 58	Claw presence vs absence
Character 59	Claw size described in relation to distal serrae of ray 4b
Character 60	Hook shape, crescent versus sickle shape (Kallman et al. 2004)
Character 61	Ramus shape around the blade
Character 62	Shape of ray 4a, four categories: from totally straight to curved in shape
Character 63	Spine angle of ray 3
Character 4	Distal serrae on ray 4b
Character 5	Well‐formed hook on ray 5a
Character 6	Granular tissue on the dorsal part of the hook on ray 3
Character 7	Subdistal spine on ray 3
Character 8	Size of segments of the distal ramus of ray 4a
Character 9	Subdistal serrae on ray 4b
Character 39	Black or darkly pigmented gonopodium
Nongonopodial traits	
Character 1	Sword
Character 2	Sword consisting exclusively of unbranched rays
Character 3	Upturned sword
Character 10	Head bump
Character 13	Elongated ventral caudal fin rays
Character 15	Growth rate
Character 16	Allometric growth of sword
Character 18	Dusky band continuous with dorsal pigment of sword
Character 19	Proximal dorsal pigmentation of the sword
Character 20	Distal dorsal sword pigment
Character 21	Grave spot
Character 22	Ventral margin of caudal fin and sword densely edged by melanophores
Character 23	Yellow and orange carotenoid sword pigmentation
Character 25	Drosopterin
Character 26	Sex‐linked red and yellow pattern
Character 30	Two or more rows of red lateral marks
Character 31	Multiple lateral stripes
Character 32	Solid mid‐lateral stripe at birth
Character 33	Vertical bars
Character 34	Body bicolored
Character 35	Dark subdermal dashes of pigment
Character 36	Two or more oblique lines behind pectoral base
Character 37	Mid‐dorsal spots
Character 38	Dorsal fin with dark marginal pigment and a sub‐basal row of dark spots on the inter‐radial membrane
Character 40	Caudal blotch
Character 41	Spotted caudal
Character 42	Carbomaculatus
Character 43	Alleles at the tailspot locus

Characters 58–63 were described in the present study. Characters 4–9, 39 were described by Marcus and McCune (1999) (original numbering of characters as per Marcus and McCune (1999) was maintained for consistency and characters described here were given unique numbers). Descriptions of nongenital characters used in the analyses of rates and modes of evolution, characterized by Marcus and McCune (1999).

### Comparison of evolutionary rates and fitting of models of trait evolution

In the first set of analyses, we aimed to compare the different suites of traits (i.e., gonopodial and nongonopodial) in terms of evolutionary rates and modes of trait evolution. All phylogenetic comparative analyses were performed using the best supported tree in Jones et al. (2013) as a reference tree. This reference tree is based on a set of RAD markers and was estimated using maximum likelihood. This tree was transformed into an ultrametric tree using the *chronopl* function in the R package *ape* (Sanderson 2002), with smoothing parameter set to 1. Then, based on the multistate character datasets (gonopodial and nongonopodial), we computed a matrix of pairwise Gower's distances (Gower 1971) between species using the R package *cluster* (Maechler et al. 2014) and restricted the analyses to the traits scored in at least half of the species (we note that some species were not scored for all traits in the previously published data utilized here) (all traits listed in Table 1 were included in these analyses). Next, we performed a principal coordinates analysis on each of these two matrices, retaining the score of each species on the first principal coordinate (accounting for 51.02% of total variation in the case of gonopodial‐related traits and 55.54% in the other set of traits) as a univariate measure of trait variation for the subsequent univariate analyses. We tested for the presence of phylogenetic signal in the multivariate datasets comprising the scores along all the principal coordinate axes for the two datasets (gonopodial and nongonopodial). This was accomplished using a method recently proposed by Adams (2014), which consists of a generalization of Blomberg's *K* statistic (Blomberg et al. 2003) to multivariate data and whose significance is tested through a permutational procedure (1000 permutations in our case; see Table 2 for a summary of all analyses conducted in this study).

**Table 2:** Overview of all analyses and results

Dataset	Test (verbal)	Test (statistical)	Result
PCoA scores from multistate characters – gonopodial traits	Phylogenetic signal	Multivariate generalization of Blomberg's *K*	*K*_mult_ = 0.56, *P* < 0.0001
	Fitting of evolutionary models	AICc and likelihood ratio test	Brownian motion (see Table 3)
PCoA scores from multistate characters – nongonopodial traits	Phylogenetic signal	Multivariate generalization of Blomberg's *K*	*K*_mult_ = 0.27, *P* = 0.0014
	Fitting of evolutionary models	AICc and likelihood ratio test	Brownian motion (see Table 3)
PCoA scores from multistate characters – gonopodial and nongonopodial traits	Comparison of evolutionary rates between sets of traits	Adams’ method on PCoA1 scores for each set of traits	*P* = 0.48
PCoA scores from multistate characters – putatively sexually selected traits	Phylogenetic signal	Multivariate generalization of Blomberg's *K*	*K*_mult_ = 0.41, *P* = 0.02
	Fitting of evolutionary models	AICc and likelihood ratio test	Brownian motion (see Table 3)
PCoA scores from multistate characters – putatively nonsexually selected traits	Phylogenetic signal	Multivariate generalization of Blomberg's *K*	*K*_mult_ = 0.29, *P* = 0.03
	Fitting of evolutionary models	AICc and likelihood ratio test	Brownian motion (see Table 3)
PCoA scores from multistate characters – putatively sexually selected and nonselected traits	Comparison of evolutionary rates between sets of traits	Adams’ method on PCoA1 scores for each set of traits	Sexually selected Robs = 2.60 Nonsexually selected Robs = 0.72 *P* = 0.002
PCoA scores from multistate characters – gonopodial and putatively sexually selected traits	Comparison of evolutionary rates between sets of traits	Adams’ method on PCoA1 scores for each set of traits	Sexually selected Robs = 2.60 Nonsexually selected Robs = 0.27 *P* < 0.001
Linear measurements on putative holdfast gonopodial features	Effect of waterflow on gonopodial morphology while accounting for phylogeny	Phylogenetic generalized least‐squares	*P* = 0.51
	Effect of waterflow on gonopodial morphology while accounting for phylogeny	Partial Mantel test keeping the matrix of patristic distances constant	*r* = 0.10, *P* = 0.24
	Correlation of ability to hybridize in the wild and gonopodial morphology, accounting for phylogeny	Partial Mantel test keeping the matrix of patristic distances constant	*r* = −0.07, *P* = 0.12
	Correlation of ability to hybridize (both in the wild and in the laboratory) and gonopodial morphology, accounting for phylogeny	Partial Mantel test keeping the matrix of patristic distances constant	*r* = −0.20, *P* = 0.004
	Correlation between existence in sympatry and gonopodial morphology, accounting for phylogeny	Partial Mantel test keeping the matrix of patristic distances constant	*r* = −0.03, *P* = 0.68

Next, to determine the evolutionary dynamics of both the gonopodial and nongonopodial trait sets, eight models were fitted and the rates of trait evolution were compared between the two sets of traits (Adams 2013). We used Adams’ (2013) method to compare the evolutionary rates between the first principal coordinate computed on the distance matrix based on gonopodial traits, and the first principal coordinate based on the other traits. We employed the R package *geiger* (Harmon et al. 2008) to fit different evolutionary models on each of the two principal coordinates. To identify the best‐fitting model, a model selection procedure was used. First, a likelihood ratio test was performed to compare a Brownian motion model (i.e., a random walk model with a constant rate of trait evolution; Felsenstein 1973) with a model of white noise to determine whether a phylogenetic model of trait variation represented a significant improvement over a model of random noise. Then, as the Brownian motion model was significantly better in both cases, the other models available in the function *fit Continuous* were fitted and compared to the Brownian motion model using a likelihood ratio test. These comprise the Ornstein–Uhlenbeck model (which is a random walk with an optimum in phenotypic space, toward which the evolution of the trait is “pulled”; Butler and King 2004), an early‐burst model (where evolutionary rates increase or decrease exponentially through time; Harmon et al. 2010), a trend model (where evolutionary rates increase or decrease linearly through time), and three models (lambda, kappa, and delta) based on tree transformations (Pagel 1999). The lambda model transforms the tree according to a parameter lambda, which ranges between zero (star‐like phylogeny, which implies that the evolution of the trait is not reflected by the phylogeny) and one (equivalent to a Brownian motion model). The kappa model differentially “stretches” longer and shorter branches; in its default implementation in *geiger*, it is a punctuational model of evolution, with values bounded to be comprised between zero (punctuational model, where the amount of evolution is independent of branch length) and one (no differential “stretching” of branches). In the delta model, based on a scaling of the path lengths, the rates of evolution can increase or decrease over time. When models fitted using default options in *fitContinuous* contained estimated parameters at their default bounds, the model was fit again increasing the range of the parameter used by the *fitContinuous* function. Among the models that fitted significantly better than the Brownian motion model (if any), the best was chosen using the version of the Akaike's Information Criterion (AIC; Akaike 1973) corrected for small sample sizes (AICc; Hurvich and Tsai 1989).

With the aim of conducting a preliminary investigation of whether sexual selection is acting on gonopodial traits, we implemented the same analyses described above to compare the rates and modes of evolution in gonopodial traits and a subset of nongonopodial traits. We compared gonopodial traits with nongonopodial traits reasonably known to be under sexual selection (vertical bars and growth rate, e.g., Ryan and Causey 1989; Morris et al. 2003; Lampert et al. 2010) and for which data are available. We do not include the sword trait (known to be preferred by females) in this subset because the evolution of the sword involves a variety of factors. For example, in some species, this trait has been lost (*Xiphophorus maculatus* and *Xiphophorus variatus*); however, females of both species prefer males with a sword; therefore, it is difficult to accurately reflect this scenario in a presence/absence matrix, for example. We additionally compared this subset of nongonopodial traits putatively under sexual selection with a subset of nongonopodial traits where the selection mechanisms acting are unknown to date (head bump, multiple lateral stripes, solid mid‐lateral stripe at birth, body bicolored, dark subdermal dashes of pigment, two or more oblique lines behind pectoral base; Table 2). This is a preliminary investigation as to date most morphological traits differentially exhibited among *Xiphophorus* species are yet to be identified as being under selection or evolving neutrally.

### Habitat, reproductive character displacement, and gonopodial morphology

To determine whether the variation in specific gonopodial traits is correlated with habitat type, that is, sites with different water flow regimes such as ponds versus flowing rivers, we used habitat data descriptions from all existing studies where water flow has been characterized for *Xiphophorus* habitats (Rosen 1960; Rauchenberger et al. 1990; Meyer and Schartl 2003; Kallman et al. 2004; Kallman and Kazianis 2006; Jones et al. 2012), as well as from unpublished data collected and verified over 35 years of regular field studies (M. Schartl, unpublished data). We note that in some instances although different species have been recorded to inhabit the exact same rivers or streams, they have also been repeatedly observed to prefer different microhabitats of those waterways (M. Schartl, unpublished data). For example in the habitats where *Xiphophorus kallmani* and *Xiphophorus milleri* predominantly occur, the swordtails (*X. kallmani*) are always seen in the middle of the stream where the current is high, and they also court in this habitat (MS, pers. obs.). In contrast, the platyfish (*X. milleri*) are only found in the calm regions of the streams, generally close to the shore and under plants (MS, pers. obs.). The same holds true for *X. variatus* and the northern swordtails. In such cases, species repeatedly recorded in the faster‐flowing regions of rivers or streams were categorized as occurring in flowing habitat types, whereas species repeatedly recorded close to the banks and under plants in slower‐flowing regions of the waterways were categorized as occurring in still‐water habitats. We categorized all known habitat types as either flowing or still water and then used phylogenetic comparative methods to test for morphological differences between habitat types in traits deemed likely to be influenced by water flow (due to the fact that they are external structures on the gonopodium). Of the major clades, the claw character is present in 16 of 17 species from the two clades typified by flowing water environments, while the claw is present in only 1 of 9 species from the clades most commonly in still‐water environments (Fig. S1). We measured a further set of five morphometric traits on the putative holdfast traits, the claw and *serrae* (Fig. S2, these are linear measurements, different from the multistate gonopodial characters used as starting data above), computed species means, and adjusted for allometric variation using standard length (sample mean). We chose to utilize the claw and *serrae* for these analyses as these features are on the external part of the gonopodium and may have holdfast functions and contribute to copulatory compatibility. All the subsequent phylogenetic comparative analyses are based on the same ultrametric tree described above for the analyses using multistate characters as starting data.

We tested for phylogenetic signal, that is, the tendency for evolutionary‐related organisms to resemble each other (Blomberg et al. 2003), in the morphometric traits on the putative holdfast traits using both a Mantel test and the adaptation of Bloomberg's *K* to multivariate data (Adams 2014). The Mantel test was used to test the significance of the correlation of allometry‐adjusted pairwise Euclidean morphometric distances with the matrix of patristic distances obtained from the phylogenetic tree: The same phylogeny was used for Adams’ method.

We used phylogenetic generalized least‐squares method (Grafen 1989; Martins and Hansen 1997; Garland and Ives 2000; Rohlf 2001) to take into account phylogenetic nonindependence when comparing habitat types using the five morphometric measurements as dependent variables. For phylogenetic generalized least‐squares method, we used the expected covariance matrix under a Brownian motion model (with gamma parameter set to 1, obtained in *ape*) as the error covariance matrix. To ensure the consistency between the analyses here and those detailed below for tests of reproductive character displacement, we also obtained pairwise interspecific Euclidean morphometric distances based on the five morphometric traits (Fig. S2) after they had been subjected to a multivariate regression‐based allometric adjustment. We then used a partial Mantel test (Smouse et al. 1986; Oden and Sokal 1992) to test for the correlation between these distances and a binary matrix indicating whether two species live in the same environment or not. To account for phylogenetic nonindependence, we kept the matrix of pairwise patristic distances constant.

Additionally, we asked whether genital evolution is influenced by the avoidance of interspecific hybridization. We addressed this question by comparing the differences in gonopodia of species pairs known to hybridize or not in nature and the laboratory. We asked whether or not those pairs that are sympatric in nature have more pronounced differences in gonopodial structure than pairs that are allopatric in nature. We utilize extensive interspecific hybridization records (both under laboratory conditions, Schartl et al. unpublished, and naturally hybridizing species, summarized in Kallman and Kazianis (2006)], as well as species geographical distribution information including sympatric and allopatric data (Tables S3, S4). We investigated sympatry and hybridization using, as outlined above, partial Mantel tests. These tests were implemented because sympatry and hybridization events can be expressed only as a property of species pairs and we could therefore not use the phylogenetic generalized least‐squares method to test for difference in the five morphometric traits. Specifically, we tested for the correlation between the matrix of pairwise morphometric distances (after allometric correction) and a binary matrix reflecting, respectively, if each pair of species lived in sympatry or not, if each pair of species hybridized under laboratory conditions, and if each pair of species hybridized under both laboratory and natural conditions (see Tables S3 and S4: data compiled from Rosen 1979; Meyer 1983; Kallman et al. 2004; Kallman and Kazianis 2006; M. Schartl pers obs.). As above, the matrix of patristic distances obtained from the phylogeny of Jones et al. (2013) was used to account for phylogenetic nonindependence in all tests.

We performed the above‐mentioned set of comparative analyses (phylogenetic generalized least‐squares test for comparing water flow regimes; partial Mantel tests for assessing the correlation of morphology with hybridization and sympatry), also on gonopodium length both accounting for allometric variation (using standard length as covariate) and using raw data.

Phylogenetic comparative analyses were performed using the R (R Core Team 2013) packages *ape* (Paradis et al. 2004), *nlme* (Pinheiro et al. 2016), *vegan* (Oksanen et al. 2016), and *adephylo* (Jombart and Dray 2008). All analyses using partial Mantel tests are based on 1000 permutations.

## Results

### Trait evolution

We first compared the evolutionary rates and modes of trait evolution in different suites of traits (gonopodial and nongonopodial). We detect a significant phylogenetic signal in both gonopodial and nongonopodial suites of traits (*K*_mult_ = 0.56 and *K*_mult_ = 0.27, respectively; *P* < 0.001 in both cases). We find that the rates of trait evolution (Adams 2013) between gonopodial and nongonopodial traits are not significantly different (*P* = 0.48), and further, we find that the best‐fitting model of trait evolution for both sets of traits is a Brownian motion model (Table 3, all results found in this study are summarized in Table 2).

**Table 3:** Models fitted for gonopodial and nongonopodial traits

Model	AICc	LRT *P*‐value
Gonopodial traits PCoA1		
**Brownian motion**	−**24.78**	–
Ornstein–Uhlenbeck	−22.21	1
Early burst	−23.81	0.21
Trend	−23.60	0.24
Lambda	−22.21	1
Kappa	−22.39	0.67
Delta	−24.25	0.154
Nongonopodial traits PCoA1		
**Brownian motion**	−**18.04**	–
Ornstein–Uhlenbeck	−15.47	0.97
Early burst	−15.47	1
Trend	−15.47	0.97
Lambda	−15.47	1
Kappa	−15.51	0.83
Delta	−15.52	0.82
Sexually selected traits PCoA1		
**Brownian motion**	**26.02**	**–**
Ornstein–Uhlenbeck	28.93	0.99
Early burst	27.68	0.26
Trend	27.91	0.31
Lambda	28.93	1
Kappa	27.54	0.24
Delta	30.25	1
Nonsexually selected PCoA1		
**Brownian motion**	**3.86**	**–**
Ornstein–Uhlenbeck	4.95	0.18
Early burst	6.77	1
Trend	5.50	0.26
Lambda	6.77	1
Kappa	6.77	1
Delta	5.11	0.20

LRT *P*‐value refers to the *P*‐value obtained when performing a likelihood ratio test comparing the model against a Brownian motion model. A *P* value lower than 0.05 would indicate that the alternative model is a better fit than a Brownian motion model. Best‐fitting models are highlighted in boldface.

In an initial investigation of the potential selection mechanisms acting on the gonopodium traits, we find that the rates of trait evolution in a subset of morphological traits reasonably known to be under sexual selection are faster than the rates of trait evolution found in gonopodial traits (*P* < 0.001; Table 2). Similarly, a subset of traits for which the underlying evolutionary mechanisms are as yet unknown are found to have a slower rate of trait evolution than the putatively sexually selected subset of traits (*P* = 0.002; Table 2). We find that the best‐fitting model of trait evolution is the same for gonopodial traits and both subsets of traits (Brownian motion) (Tables 2, 3). Further, we detect a significant phylogenetic signal in both subsets of traits (putatively sexually selected traits *K*_mult_ = 0.41 and putatively nonsexually selected traits *K*_mult_ = 0.29, respectively; *P* < 0.05 in both cases; Table 2).

### Determinants of gonopodial morphology

We determined whether the variation in specific gonopodial traits is correlated with habitat type. The claw (a putatively important holdfast trait) is present in the majority of species occurring predominantly in fast‐flowing habitats, whereas it is absent in species preferring slow‐flowing habitats (Fig. 1B, Table 1, Fig. S1). Using two analyses of phylogenetic signal, we find that there is a significant phylogenetic signal (*r* = 0.22, Mantel test *P* < 0.001; Adams’ *K*_mult_ = 0.608 *P* < 0.0001) in the analyzed traits (dataset of linear measurements of putatively holdfast traits); that is, the more closely related two species are, the more similar they are as well in their gonopodial morphology. Interestingly, when using the measurement data of the claw and *serrae* (Fig. 1, Table 1, traits measured shown in Fig. S2), there are no significant differences found between habitat types in any of the comparative methods used.

In addition, we find that the correlation between sympatry and morphometric distances is not significant (both Mantel and partial Mantel, *P* > 0.05) (Table 2). This suggests that there is no evidence of patterns typically associated with reproductive character displacement (Shapiro and Porter 1989; Arnqvist 1998). We find that there is a significant negative correlation between species known to hybridize in nature and the laboratory and the analyzed morphometric distances when taking into account phylogenetic nonindependence (*r* = −0.2, partial Mantel test, *P* = 0.004). However, the correlation between morphometric measurements and hybridization under natural conditions is lower and not significant (Table 2).

## Discussion

We show that the highly variable *Xiphophorus* gonopodial structure is not evolving more rapidly than other nongenital traits in this diverse genus. While male genital morphology is variable among *Xiphophorus* species, there is no difference in evolutionary rates of change or modes of evolution when compared with nongonopodial traits. We find that a Brownian motion model is the best‐fitting model for both trait types. In a Brownian motion model, the state of a character can increase or decrease at each instant in time, and the magnitude and direction of these shifts are independent of the current state of the character and have a net change of zero (O'Meara et al. 2006). The lack of difference in rate and mode of gonopodial evolution compared to nongonopodial evolution may be explained by similar selection mechanisms acting on both trait types in *Xiphophorus* fishes. It is a common assumption that genital traits are more variable (e.g., due to sexual rather than natural selection pressures) or evolve more rapidly (e.g., where prezygotic isolation is expected to evolve faster than postzygotic isolation; Coyne and Orr 1989) than nongenital morphological traits (Arnqvist 1998; Hosken and Stockley 2004; Eberhard 2010a, 2010b). Recently, this has indeed been shown to be the case in an ecologically and morphologically highly diverse group of squamate reptiles, Caribbean *Anolis* lizards (Klaczko et al. 2015). However, the results gained here suggest that this trend may not be universal. Thus, although we find no difference in gonopodial rates of evolution compared to nongonopodial traits, the question remains: “What is driving the diversity in form of this elaborate trait?”

Utilizing morphological data gathered in this study, and already available morphological characterizations and habitat descriptions, we examined whether natural selection mechanisms, both mating and nonmating, play a role in shaping gonopodial morphology. Habitat ecology, in particular flow velocity of the water environment, may select for genital morphology that ensures the successful transfer of sperm. A shift in the breeding habitat of these fish may select for the most effective holdfast mechanisms, assuming that those mechanisms are otherwise costly. While the presence of such a trait (claw) might be related to water flow, the fine‐scale morphometric variation in holdfast traits shows no correlation with habitat type. However, future collection and analyses of more detailed habitat data for all *Xiphophorus* species will allow us to gain higher‐resolution results than those possible with the currently available data, and such results may differ from what we find here. Nonmating natural selection, such as selection for locomotor performance or the presence of predators, may also play a role in the divergence of gonopodial morphology (e.g., as was shown in poeciliid fish; Kelly et al. 2000; Langerhans et al. 2005). However, again here, we find no difference in gonopodial lengths between fast‐ and slow‐flowing habitats, while further studies are required to investigate the influence of predators. Similar to the sword in these fish, the evolution and development of the gonopodium may have little impact on a male's ability to swim (Oufiero and Garland 2007; Oufiero et al. 2012, 2014a). These results are consistent with previous studies suggesting that different mechanisms, other than habitat ecology, need to be considered as potential drivers of variation in male genital morphology (e.g., Jennions and Kelly 2002).

Further, we show that the gonopodium is unlikely to be subject to reproductive character displacement or selection against hybridization. Although our results provide evidence for the premise that species with more similar gonopodial morphologies can and *do* hybridize in the laboratory, in nature there is no evidence for the predicted outcome (i.e., that species living in sympatry show higher morphological divergence). In fact, we find no evidence for higher trait distance between species in sympatry versus allopatry. This might suggest that there are other prezygotic isolating mechanisms, such as mating behavior, acting to keep these species apart and that such traits may also be evolving faster than the differences in gonopodial morphology. Because most species in this genus hybridize in the laboratory, if not given a choice, the gonopodial traits (and female genital differences that might exist) do not provide an effective barrier to hybridization anyhow. These results are in line with one of the most important criticisms of the role of structural lock‐and‐key mechanisms in reproductive isolation in particular; that is, that species possessing dramatic differences in genital morphology can often mate and produce offspring (Robson and Richards 1936; Masly 2012). Investigations of female genital morphology among *Xiphophorus* species, and whether there is intraspecific correlated evolution of male and female genitalia, would further strengthen our understanding of the role of structural reproductive isolation (Masly 2012) in these fish. Similarly, the possibility of reproductive isolation being influenced by sensory lock‐and‐key mechanisms remains to be investigated in *Xiphophorus*. The poeciliid genus *Gambusia* by comparison, which like *Xiphophorus* exhibits much interspecific gonopodial diversity, shows significant reproductive character displacement both in the male gonopodia and in female genital morphology (Langerhans 2011). These so far contrasting results between *Xiphophorus* and *Gambusia* suggest that a diversity of selective forces are contributing to male genital variation in this family of about 280 species and about 28 genera of livebearing fishes.

Is the gonopodium a key target of sexual selection? Previous studies of livebearers suggest that sexual selection may be causal in the diversity of structures seen in the gonopodium (Langerhans 2011). The finding here of slower rates of evolution in gonopodial traits compared to a subset of nongonopodial traits thought to be under sexual selection suggests that different mechanisms might be acting on the gonopodium compared to such traits. However, the same evolutionary model (Brownian motion) was found to be the best‐fitting model for the gonopodial traits and both the putatively sexually selected subset of traits and a subset of traits where the selection mechanisms acting are not known, suggesting instead that similar evolutionary mechanisms may be acting on all these different trait sets. Comparisons with the putatively sexually selected subset of traits were necessarily based on a small subset of nongonopodial traits (due to a lack of current information driving the diversity of these traits) and would greatly benefit from studies of the underlying forces governing the diversity of form in more of the morphological traits in this genus. Thus, the preliminary inquiry conducted here into the potential role of sexual selection mechanisms on the evolution of the diverse *Xiphophorus* gonopodium has just begun to scratch the surface, and further investigations are imperative for determining more conclusively how and why sexual selection might be acting on this elaborate trait.

### Next targets of investigation

Broadly, the question of which mechanisms underlie the striking diversity of genital morphologies has received the most attention by researchers and empirical support from sexual selection theory (Eberhard 1985, 2010a; Arnqvist 1998; Hosken and Stockley 2004; Langerhans 2011). *Xiphophorus* exhibit an array of gonopodial morphologies (Fig. 2), some of which may be shaped by sexual selection processes. The armament or putative optimal holdfast traits, hooks, spines, and claws (Fig. 1, Table 1), for example, may be influenced by a combination of sperm competition, cryptic female choice, and postmating sexual conflict (Langerhans 2011). These holdfast traits appear to be key candidates in sperm competition as they may enhance insemination or postinsemination fertilization success by increasing the duration of copulation and therefore sperm transfer success, or place sperm in favorable locations in the female genitalia. Such traits might also reduce the insemination or fertilization success of rival males through removing sperm or causing injuries to female genitalia that tend to cause females to be chaste, and might be sexually antagonistic and prevent further copulations (Constanz 1984; Langerhans 2011). Further, under the postmating sexual conflict hypothesis of genital evolution, one of the main predictions is that male genital traits that increase male fitness reduce female fitness and cause females to directly benefit from rejecting some conspecific males by reducing the direct costs of unwanted inseminations. The claw, hooks, spines, and *serrae* structures in *Xiphophorus* appear to be “offensive structures,” which suggests that they might have a role in sexual conflict; again, further studies are needed to test such predictions directly (Langerhans 2011). Additionally, poeciliids are known to vary even intraspecifically in the frequency with which males utilize coercive mating tactics, such as gonopodial thrusting, and these differences can correlate with gonopodium shape and size (e.g., Farr et al. 1986, see also Ptacek and Travis 1998). It would be interesting to perform further tests to determine whether such differences in mating tactics are correlated with the differences in shape and size of the *Xiphophorus* gonopodium. If sperm competition and/or postmating sexual conflict is driving the functional morphology of the gonopodium, one would expect such unique keys to have specific lock counterparts (Eberhard 2004; Eberhard and Ramirez 2004; Jagadeeshan and Singh 2006). As suggested above, to date this has not been described for poeciliid females. However, there is evidence that female genitalia vary across populations with different expected levels of sexual coercion (Evans et al. 2013); therefore rather than functioning as a lock, the female gonopore may function to deter coercive copulations.

A likely alternative driver of elaborate male genital morphology is female choice. As suggested by Langerhans (2011) for poeciliids more generally, the distal tip of the *Xiphophorus* gonopodium is quite unusual and is likely to be the object of cryptic female choice. Cryptic female choice has been well studied in insects and spiders and is thought to influence the evolution of extraordinary male genital morphologies, and we are now beginning to understand how this might apply to poeciliids (Evans et al. 2011; Langerhans 2011). In *Drosophila*, for example, male genitalia vary radically in size and shape between closely related species, whereas female genital morphology tends to be less variable (Eberhard 1985). This variation in males is likely the result of female choice and conflict (Jagadeeshan and Singh 2006). Further, using fluorescently labeled sperm protein, it has recently been shown that *Drosophila simulans* females can alter the proportion of conspecific and heterospecific sperm stored (Chippindale 2013; Manier et al. 2013). Specific functional tests and comparisons between the roles of different sexual selection pressures, and particularly investigating the role of female choice, are important next steps in unraveling exactly how highly variable male genital morphology arises, and also whether these traits may be key to species diversification in poeciliid fishes.

## Conclusions

In this study, we have shown that there are elaborate interspecific differences in male genital morphology in the genus *Xiphophorus*. We provide evidence for no differences in evolutionary rates or modes of evolution in genital and nongenital traits in these fish, suggesting a commonality in the forces shaping gonopodial and nongonopodial traits. Natural selection mechanisms, both mating and nonmating, do not appear to be driving the diverse *Xiphophorus* gonopodial morphology. We find inconsistent evidence that the putative holdfast features of the male genital organ are affected by water flow, a candidate ecological selective mechanism in aquatic environments. Additionally, the finding that interspecific divergence in gonopodial morphology is not significantly higher between sympatric species, than between allopatric species, would seem to argue against the hypothesis that genital evolution plays a major role in speciation resulting in reproductive character displacement. Our results also indicate that gonopodial traits may be evolving at a slower rate than a subset of nongonopodial traits thought to be under sexual selection. However, further investigations of these genital structures are the important next steps in understanding if and how sexual selection (as opposed to more neutral evolution) may be involved in driving the evolution of the gonopodium.

## Conflict of Interests

None declared.

## Supporting information


**Figure S1.** Mirror tree depiction of the relationship between fast and slow flowing habitats (preferred) and the presence of the putative hold fast trait, the claw. Open circles indicate no data is available.

**Figure S2.** Morphometric traits measured on the claw and serrae of the gonopodium of all *Xiphophorus* species.

**Table S1.** Specimens by origin and species.

**Table S2.** Raw scores of all gonopodial traits used in this study.

**Table S3.** Summary of sympatric, allopatric and naturally hybridizing species pairs in the genus *Xiphophorus*.

**Table S4.** Summary of species known to hybridize in the laboratory.
