# Supplementary material for: Evolution of the elaborate male intromittent organ of Xiphophorus fishes
Source: Ecol Evol. 2016 Sep 17;6(20):7207–20. doi: 10.1002/ece3.2396 (PMC5114703; doi:10.1002/ece3.2396)
Supplement: Supplementary file 1 — Figure S1. Mirror tree depiction of the relationship between fast and slow flowing habitats (preferred) and the presence of the putative hold fast trait, the claw. Open circles indicate no data is available. [file ECE3-6-7207-s001.pdf]

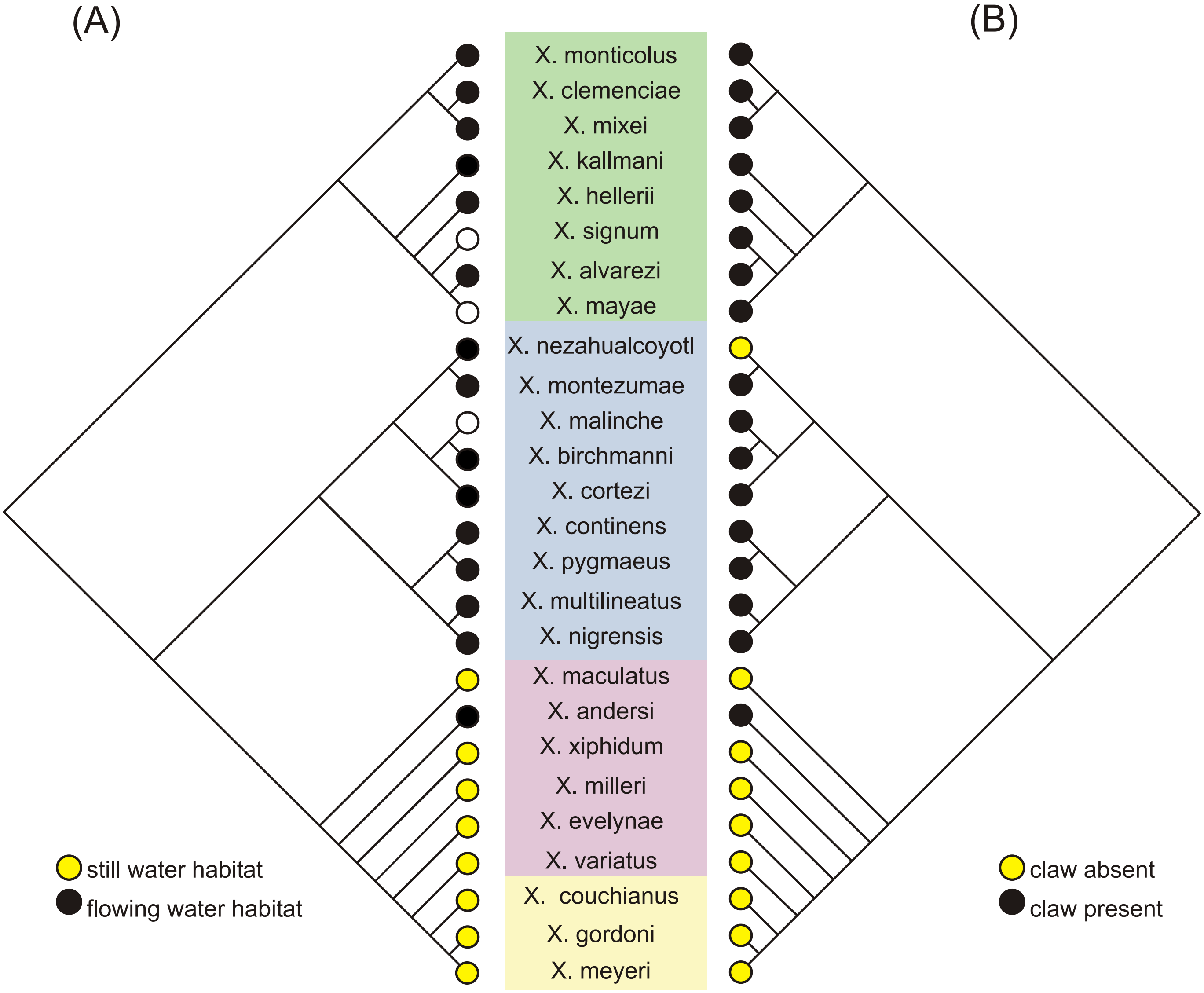

(A)
(B)
X. monticolus
X. clemenciae
X. mixei
X. kallmani
X. hellerii
X. signum
X. alvarezi
X. mayae
X. nezahualcoyotl
X. montezumae
X. malinche
X. birchmanni
X. cortezi
X. continens
X. pygmaeus
X. multilineatus
X. nigrensis
X. maculatus
X. andersi
X. xiphidum
X. milleri
X. evelynae
X. variatus
X. couchianus
X. gordoni
X. meyeri
still water habitat
flowing water habitat
claw absent
claw present
