# Supplementary figures and images for: Evolution of the elaborate male intromittent organ of Xiphophorus fishes
Source: Ecol Evol. 2016 Sep 17;6(20):7207–20. doi: 10.1002/ece3.2396 (PMC5114703; doi:10.1002/ece3.2396)

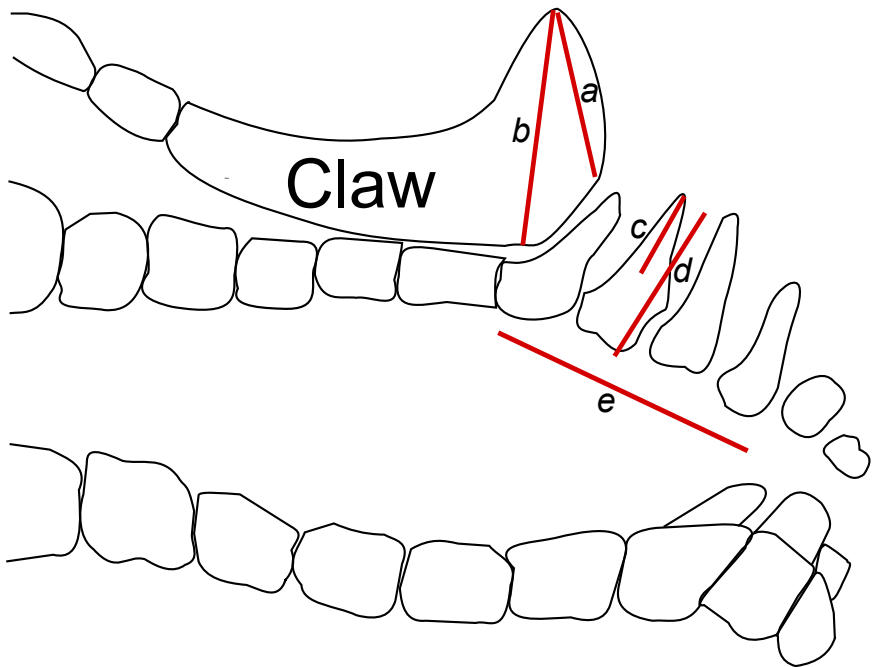

Supplement: Supplementary file 2 — Figure S2. Morphometric traits measured on the claw and serrae of the gonopodium of all Xiphophorus species. [file ECE3-6-7207-s002.pdf]
