# Supplementary material for: Evolution of the elaborate male intromittent organ of Xiphophorus fishes
Source: Ecol Evol. 2016 Sep 17;6(20):7207–20. doi: 10.1002/ece3.2396 (PMC5114703; doi:10.1002/ece3.2396)
Supplement: Supplementary file 3 — Table S1. Specimens by origin and species. [file ECE3-6-7207-s003.docx]

| **Species** | **Sampling site** | **No. of individuals** |
| --- | --- | --- |
| *X. alvarezi* | Rio Dolores | 5 |
| *X. andersi* | Río Atoyac | 3 |
| *X. birchmanni* | Río Axtlapexco | 1 |
| *X. clemenciae* | Puente Chino Luiz | 4 |
| *X. continens* | Ojo Frío | 2 |
| *X. cortezi* | Río Axtla | 3 |
| *X. couchianus* | Apodaca | 4 |
| *X. evelynae* | Tecolutla | 1 |
| *X. gordoni* | Santa Tecla | 3 |
| *X. hellerii* | Río Lancetilla | 4 |
| *X. kallmani* | Laguna Catemaco | 2 |
| *X. maculatus* | Río Grijalva, Cuatro Hermanos, Río Jamapa | 7 |
| *X. malinche* | Arroy Xontla | 2 |
| *X. mayae* | Río Dulce | 2 |
| *X. meyeri* | Melchor Musquiz | 3 |
| *X. milleri* | Laguna Catemaco | 4 |
| *X. mixei* | Rio El Sol | 1 |
| *X. montezumae* | Cascadas de Tamasopo | 2 |
| *X. monticolus* | El Tejon | 5 |
| *X. multilineatus* | Río Coy | 4 |
| *X. nezahualcoyotl* | Río El Salto | 3 |
| *X. nigrensis* | Nacimiento de Choy | 1 |
| *X. pygmaeus* | Río Axtla | 3 |
| *X. signum* | Río Chajmaic | 5 |
| *X. variatus* | Cuidad Mante | 4 |
| *X. xiphidium* | Río Purificación | 1 |
| *Gambusia holbrooki* | Everglades, Florida | 4 |
| *Heterandriae formosa* | Everglades, Florida | 1 |
| *Priapella intermedia* | Río La Lana | 3 |
