# Supplementary material for: Evolution of the elaborate male intromittent organ of Xiphophorus fishes
Source: Ecol Evol. 2016 Sep 17;6(20):7207–20. doi: 10.1002/ece3.2396 (PMC5114703; doi:10.1002/ece3.2396)
Supplement: Supplementary file 7 [file ECE3-6-7207-s007.docx]

**Supporting Information**

**Figure S1.** Mirror tree depiction of the relationship between fast and slow flowing habitats (preferred) and the presence of the putative hold fast trait, the claw. Open circles indicate no data is available.

**Figure S2.** Morphometric traits measured on the claw and serrae of the gonopodium of all *Xiphophorus* species.

**Table S1.** Specimens by origin and species.

**Table S2.** Raw scores of all gonopodial traits used in this study.

**Table S3.** Summary of sympatric, allopatric and naturally hybridising species pairs in the genus *Xiphophorus.*

**Table S4.** Summary of species known to hybridise in the laboratory.
